# Mean CD4 cell count changes in patients failing a first-line antiretroviral therapy in resource-limited settings

**DOI:** 10.1186/1471-2334-12-147

**Published:** 2012-06-28

**Authors:** Alexandra Calmy, Eric Balestre, Fabrice Bonnet, Andrew Boulle, Eduardo Sprinz, Robin Wood, Eric Delaporte, Eugène Messou, James McIntyre, Kamal Marhoum El Filali, Mauro Schechter, N Kumarasamy, David Bangsberg, Patrick McPhail, Stefaan Van Der Borght, Carlos Zala, Matthias Egger, Rodolphe Thiébaut, François Dabis

**Affiliations:** 1 HIV Unit, Geneva University Hospital, Geneva, Switzerland; 2 Univ. Bordeaux, ISPED, Centre Inserm U897- Epidemiologie-Biostatistique, F-33000, Bordeaux, France; 3 INSERM, ISPED, Centre Inserm U897- Epidemiologie-Biostatistique, F-33000, Bordeaux, France; 4 Service de Médecine Interne et Maladies Infectieuses, Centre Hospitalier Universitaire (CHU), Bordeaux, France; 5 School of Public Health and Family Medicine, University of Cape Town, Cape Town, South Africa; 6 South Brazil HIV Cohort, Hospital de Clinicas de Porto Alegre, Porto Alegre, Brazil; 7 Gugulethu ART Programme, Gugulethu, South Africa; 8 Institut de Recherche et de Développement (IRD), Montpellier, France; 9 Centre de Prise en Charge, de Recherche et de Formation sur le VIH/SIDA (CePReF), Abidjan, Côte d’Ivoire; 10 Perinatal HIV Research Unit, Operational Research on ART (OPERA), Soweto, South Africa; 11 Service des Maladies Infectieuses, Hôpital Ibn Rochd, Casablanca, Maroc; 12 Rio HIV Cohort, Rio de Janeiro, Brazil; 13 YRG Centre for AIDS Research and Education, Chennai, India; 14 Immune Suppression Syndrome Clinic (ISS), Mbarara, Uganda; 15 Helen Joseph Hospital Themba Lethu Clinic, Johannesburg, South Africa; 16 Heineken International Health Affairs, Amsterdam, Netherlands; 17 Prospective Evaluation in the Use and Monitoring of Antiretrovirals in Argentina (PUMA), Buenos Aires, Argentina; 18 Institute of Social and Preventive Medicine (ISPM), University of Bern, Bern, Switzerland; 19 4 rue Gabrielle Perret Gentil, Geneva University Hospital, 1211, Geneva 4, Switzerland

**Keywords:** HIV-1, CD4 count, CD4 slope, HIV-RNA threshold, Resource limited settings

## Abstract

**Background:**

Changes in CD4 cell counts are poorly documented in individuals with low or moderate-level viremia while on antiretroviral treatment (ART) in resource-limited settings. We assessed the impact of on-going HIV-RNA replication on CD4 cell count slopes in patients treated with a first-line combination ART.

**Method:**

Naïve patients on a first-line ART regimen with at least two measures of HIV-RNA available after ART initiation were included in the study. The relationships between mean CD4 cell count change and HIV-RNA at 6 and 12 months after ART initiation (M6 and M12) were assessed by linear mixed models adjusted for gender, age, clinical stage and year of starting ART.

**Results:**

3,338 patients were included (14 cohorts, 64% female) and the group had the following characteristics: a median follow-up time of 1.6 years, a median age of 34 years, and a median CD4 cell count at ART initiation of 107 cells/μL. All patients with suppressed HIV-RNA at M12 had a continuous increase in CD4 cell count up to 18 months after treatment initiation. By contrast, any degree of HIV-RNA replication both at M6 and M12 was associated with a flat or a decreasing CD4 cell count slope. Multivariable analysis using HIV-RNA thresholds of 10,000 and 5,000 copies confirmed the significant effect of HIV-RNA on CD4 cell counts both at M6 and M12.

**Conclusion:**

In routinely monitored patients on an NNRTI-based first-line ART, on-going low-level HIV-RNA replication was associated with a poor immune outcome in patients who had detectable levels of the virus after one year of ART.

## Background

Antiretroviral Therapy (ART) has undoubtedly changed lives, both in resource-rich and in resource-limited settings (RLS). While a large majority of patients on antiretroviral (ARV) drugs achieve sustained virological suppression, some experience a treatment failure eventually leading to treatment change. Treatment switches to second–line ARV regimens in RLS occur at a lower rate than expected due to several constraints: limited access to virological monitoring, limited availability of suitable ARV drugs, pill burden, toxicity and cost, thus limiting the use of second-line ART regimens even in failing patients [1-4]. Overall, it is estimated that second–line ART accounts for <5% of total ARV use in RLS at the present time [5].

Currently, most treatment failures are diagnosed by a CD4 cell count decrease, or by a clinical event as per the 2006 World Health Organization (WHO) guidelines [6]. Cohort analyses suggested that non-virological criteria do not accurately predict virological failure [7,8].

Two randomised trials, (*Home-Based AIDS Care* [HBAC] and *Development of Antiretroviral Therapy in Africa* [DART] [9,10], have addressed the question of the efficacy of monitoring strategies in RLS. A meta-analysis of these two trials concluded that clinical monitoring alone (compared to combined immunological and clinical monitoring or to combined virological, immunological, and clinical monitoring) resulted in unnecessary treatment switches, disease progression, and increased mortality [11]. These results were not confirmed by a recent study conducted in Cameroon in which no differences between laboratory-based or clinical strategies were demonstrated at 24 months [12]. Another clinical trial in Thailand failed to demonstrate a significant difference in clinical failure among patients whose switching decision was based on CD4 cell counts versus HIV-RNA at 3 years [13]. Nevertheless, newer WHO recommendations do recommend using HIV-RNA plasma viral load (HIV-RNA) to confirm a suspected treatment failure [14].

The optimal threshold of HIV-RNA and the optimal frequency of HIV-RNA determination that best inform antiretroviral switches in RLS are still being debated. In Europe, Australia and the USA, most clinicians continue to rely on the approach in which a virological failure is defined by an HIV-RNA level above the detection threshold; and complete viral suppression is the goal of ART, with HIV-RNA level being monitored every three to six months. Thus, the American guidelines and the guidelines from various European countries all propose that HIV-RNA detection is the essential tool by which to monitor the need to switch ART [15,16].

This strategy of maximal viral suppression may be less appropriate in a context in which the second-line treatment may be considered as the last chance for a suppressive regimen. The PLATO study suggested that ART regimen that maintains a viral load of <10,000 copies/ml was not associated with an appreciable CD4 cell count decline in triple-class experienced patients after a median follow-up period of two years [17]. Similarly, an Italian cohort study on 3023 patients over a median clinical follow-up time of 46 months showed that the risk of clinical progression was lower in patients with moderate viremia (<10,000 copies/ml), compared with patients with higher HIV-RNA levels [18].

We aimed to investigate the effect of on-going HIV-RNA replication at low levels in routinely monitored patients on a non-nucleoside reverse transcriptase inhibitor (NNRTI) based first-line ARV regimen in RLS. For this purpose, we analysed changes in CD4 cell counts in patients with an incomplete virological response or with a virological failure to two ARV drug classes.

## Methods

### The ART-LINC of IeDEA collaboration

The Antiretroviral Therapy in Low-Income Countries (ART-LINC) Collaboration is a network of antiretroviral treatment programs in Africa, Latin America and Asia. This project has been reorganised in the International epidemiological Database to Evaluate AIDS (IeDEA) initiative ( http://www.iedea-hiv.org/). Data sources, the merging of data and quality control procedures have been described elsewhere [19]. Patient information in the pertinent database is de-identified. This research has been carried out in compliance with the Helsinki Declaration and Institutional review boards in all countries approved the analysis of routinely collected programme data at all sites.

### Eligibility criteria and definitions

For this analysis, we selected from the IeDEA database all HIV-1 infected naïve adults (16 years or older) with gender and date of ART initiation known, treated with a first–line ARV combination including two nucleoside analogues (NRTIs) and one protease inhibitor (PI) or one NNRTI. Patients needed to have an HIV-RNA measurement at least six and 12 months after ART initiation (plus or minus three months) and to have at least one CD4 cell count measure between six months (or within the three months before) and 18 months following ART commencement. We excluded all patients with undetectable HIV-RNA levels (<500 copies/ml) at ART initiation.

We examined the data for these subjects up to 18 months after ART initiation or at the date of switch to a second-line ARV regimen. Second-line ART was defined by at least one therapeutic class modification and at least one NRTI modification. A patient was considered lost to follow-up if the patient was not known to have died and the time interval between the last date he (she) was known to be alive and the closure date of cohorts (i.e., date of last record) was >6 months.

### Statistical analysis

We assessed the CD4 cell count response to treatment between six and 18 months after ART initiation, using linear mixed models. The changes in CD4 cell count were modeled with two slopes: the first slope was for the six to 12 months after ART initiation and the second slope was for the 12 to 18 months after ART initiation. An intercept at the initial time of the analysis (six months after ART initiation) was also included in the linear mixed models. To take into account the correlation of the repeated measurements within each patient, the parameters were allowed to vary from one patient to another through the random intercept and the two random slopes. An unstructured covariance matrix was used for random effects, allowing a correlation between the individual baseline level and the slopes. To avoid any collider bias CD4 count changes were not adjust for initial CD4 count as fixed effects. We considered (*a priori*) three HIV-RNA thresholds (500 [lower limit], 5,000 and 10,000 copies/ml [upper limit]) 6 and 12 months after ART initiation.

Linear mixed models were adjusted for age at the time of ART initiation, gender, year of starting ART and clinical stage at starting ART. These adjustment factors were included as main effects to adjust the intercept at six months and as interactions with time to adjust CD4 slopes. HIV-RNA measured both at six and 12 months after ART initiation were entered as covariates in the model to estimate the effect of HIV-RNA on the first and the second CD4 count slope respectively. We considered 40 year old (or older) women starting ART in 2004 or later at clinical stage A, B (CDC classification) or I, II (WHO classification) and with a viral load at M6 and M12 <500 copies/mL as the reference group. We compared mixed models using these HIV-RNA thresholds with the Akaike Information Criterion (AIC).

All analyses were performed using SAS version 9.1 (SAS Institute, Cary, North Carolina, USA).

## Results

### Population

**Figure 1:**
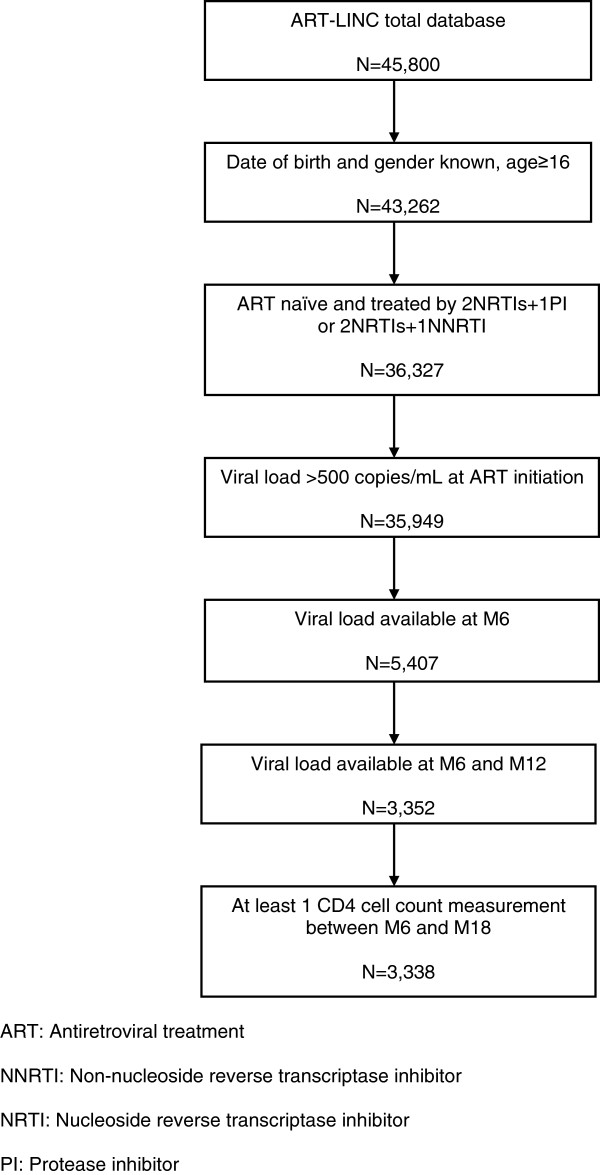
**Study flow chart.** ART-LINC of IeDEA Cohort Collaboration.

We included 3,338 patients from 14 different cohorts in this analysis (Figure 1). The number of patients per cohort ranged from 46 to 891, with nine cohorts from sub-Saharan Africa (accounting for 80.1% of the overall study population), three cohorts from Latin America (14.7%), one cohort from Morocco and one from India. The patient group had the following characteristics: 2,139 patients were female (64.1%), the median age was 34 years (inter-quartile range (IQR) = 30-41); 32.3% of the patients were in WHO stage I or II at ART initiation; 2,070 (62.0%) patients initiated their first-line ART in or after 2004; and for 96.0% of the patients, the ART regimen included an NNRTI and two NRTIs as per WHO recommendations. The follow-up characteristics of the patient group are as follows: the total number of person–years of follow-up was 6,778; the median follow-up time was 1.6 years (IQR = 1.2-2.4); 3,039 patients (91.0%) were still followed up 12 months after ART initiation and 1,995 (59.8%) were still followed up at 18 months; and 44 (1.3%) and 325 (9.7%) patients were lost to follow-up at 12 and 18 months, respectively (Table 1).

**Table 1:** Patient characteristics at ART initiation, and during follow-up (ART-LINC of IeDEA, 2008, n = 3,338)

**Characteristics at ART initiation**	**N**	
Women (%)	2,139	(64.1)
Median age (IQR*)	34.0	(30–41)
Clinical stage (%)		
WHO I/II or CDC A/B	1,078	(32.3)
WHO III	977	(29.3)
WHO IV or AIDS	1,019	(30.5)
Unknown	264	(7.9)
Year of starting ART (%)		
1997 to 2001	351	(10.5)
2002 to 2003	917	(27.5)
2004 to 2005	1,629	(48.8)
2006 to 2007	441	(13.2)
First ART regimen (%)		
2NRTIs + 1NNRTI	3,205	(96.0)
2NRTIs + 1PI	133	(4.0)
Median CD4 cell count (IQR)	107	(46–179)
HIV-RNA (%)		
[500–10,000[ copies/mL	170	(5.1)
≥10,000 copies/mL	1,942	(58.2)
Unknown	1,226	(36.7)
**Characteristics 6 months after ART initiation**		
Median CD4 cell count (IQR)	228	(158–325)
HIV-RNA (%)		
<500 copies/mL	3,038	(91.0)
[500–10,000[ copies/mL	155	(4.6)
≥10,000copies/mL	145	(4.4)
**Characteristics 12 months after ART initiation**		
Median CD4 cell count (IQR)	276	(192–383)
HIV-RNA (%)		
<500 copies/mL	2,969	(89.0)
[500–10,000[ copies/mL	187	(5.6)
≥10,000 copies/mL	182	(5.4)
Median follow-up in years (IQR)	1.6	(1.2-2.4)
Median number of CD4 measurements (IQR)	2	(2–3)
Deaths at M18 (%)	18	(0.5)
Lost to follow-up at M18 (%)	325	(9.7)

* IQR = interquartile range.

ART: Antiretroviral treatment.

NNRTI: Non nucleoside reverse transcriptase inhibitor.

NRTI: Nucleoside reverse transcriptase inhibitor.

PI: Protease inhibitor.

### CD4 cell count and HIV-RNA: Data description

A majority of patients (83.9%) had three or more HIV-RNA determinations during the study period. Similarly, 87.8% had at least three CD4 cell counts and 46.2% had a greater number of measurements up to 18 months. Median HIV-RNA level (log10) at ART initiation was 5.07 (IQR 4.53-5.54) and the median CD4 cell count was 107 cells/mm^3^ (IQR = 46-179); 3,038, 2,969 and 814 individuals had an HIV-RNA level below the threshold of 500 copies/ml at M6, M12 and M18, respectively, after starting ART (91.0%, 89.0% and 87.7% of all available viral load measurements, respectively).

Among patients initiating ART with HIV-RNA levels above 10,000 copies/ml, 91.7% (n = 1,780) and 90.0% (n = 1,747) achieved HIV-RNA levels below 500 copies/ml six months and 12 months, respectively, after ART commencement. Among patients with undetectable (<500 copies/ml) HIV-RNA levels (n = 3,038) at M6, 93.0% (n = 2,825) remained with an undetectable HIV-RNA level and 3.0% (n = 92) had an HIV-RNA level ≥10,000 copies/ml at M12. Among patients with undetectable HIV-RNA levels six and 12 months after starting ART (n = 2,825), 750 (94.1% of patients with available data) remained with an undetectable HIV-RNA at M18.

### CD4 cell count and HIV-RNA longitudinal analysis

**Figure 2:**
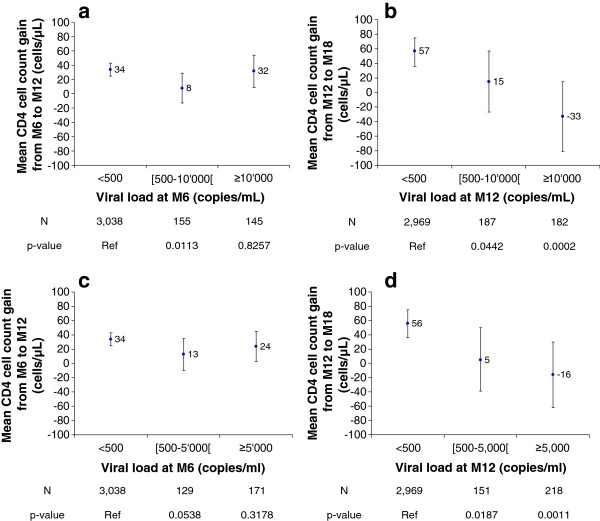
**a: Mean CD4 cell count change between M6-M12 relative to the M6 viral load (adjusted, threshold = 10,000 copies/ml). b:** Mean CD4 cell count change between M12-M18 relative to the M12 viral load (adjusted, threshold = 10,000 copies/ml). **c:** Mean CD4 cell count change between M6-M12 relative to the M6 viral load (adjusted, threshold = 5,000 copies/ml). **d:** Mean CD4 cell count change between M12-M18 relative to the M12 viral load (adjusted, threshold = 5,000 copies/ml).

We assessed the effect of the HIV-RNA level while on ART on the mean CD4 cell count slope. Figure 2-a shows the adjusted mean CD4 cell count slopes (for the 6 to 12 months period) of patients with an M6 HIV-RNA level below or above a threshold of 10,000; Figure 2-c gives these slopes using an HIV RNA threshold of 5,000. Figure 2-b shows the CD4 cell count slopes (for the 12 to 18 months period) of patients with an M12 HIV-RNA level below or above a threshold of 10,000; Figure 2-d shows the same slope for patients with a 5,000 threshold. The CD4 cell count SLOPE was not significantly different from the null value between six and 12 months following ART initiation, regardless of the HIV-RNA replication level (Figures 2-a, c). However, differences related to HIV-RNA replication levels were observed after 18 months: only patients with suppressed HIV-RNA levels at 12 months had a continuous increase in CD4 cell count, whereas those with any degree of HIV-RNA replication (either 10,000 or 5,000 copies) 12 months after ART commencement had either no increase or a decrease of their CD4 cell count slope (Figures 2-b, d).

Table 2 shows that age, sex, CDC stage, and the calendar year in which ART was initiated were all significant determinants of CD4 cell count six months after ART initiation. Moreover, we modelled the effect of several HIV RNA values at M6 and M12 following ART initiation on the CD4 cell count changes between M6 and M18; CD4 cell count increased by 90 cells (95% CI = 71; 108) per year when patients had a suppressed HIV-RNA level at both M6 and M12; in contrast, CD4 cell count decreased by 1 cell per year (CI = −51; 48) when HIV-RNA was consistently above 10,000 copies (Table 2). In addition, similar CD4 cell count changes were observed when a lower HIV-RNA threshold (5,000 copies) was chosen as a cut-off (AIC = 106669 for the 10,000 versus 106679 for the 5,000 copies threshold) [data not shown].

**Table 2:** CD4 count six months after ART initiation and CD4 changes estimated by adjusted linear mixed model, relative to the viral load six and 12 months after starting ART (HIV-RNA threshold 10,000 copies), ART-LINC of IeDEA

**Variables**	**Mean CD4 cell count at M6 (cells/μL)**	**95% CI**	**p value**
**Viral load at M6**			<0.0001
<500	289*	277;301	
[500–10,000[ (vs <500)	−3	−26;+20	
≥10,000 (vs <500)	−82	−106;-58	
**Gender**			0.0005
Male vs female	−18	−29;+8	
**Age at starting ART (years)**			0.0451
[16-30[ vs. ≥40	+13	−1;+27	
[30-35[ vs. ≥40	−3	−16;+10	
[35-40[ vs. ≥40	−7	−21;+7	
**Year of starting ART**			0.0111
Prior to 2004 vs. up to 2003	+14	+3;+25	
**Stage at starting ART**			<0.0001
III vs. A,B/I,II	−50	−62;-38	
AIDS/IV vs. A,B/I,II	−46	−58;-33	
Unknown vs. A,B/I,II	−5	−24;+14	
	**Mean CD4 cell count change from M6 to M18 (cells/μL)**		
Viral load at M6 and M12^*^			<0.0001
Viral load <500 at M6 and <500 at M12	+90	+71;+108	
Viral load <500 at M6 and [500–10,000[ at M12	+49	+7;+91	
Viral load <500 at M6 and ≥10,000 at M12	+1	−47;+49	
Viral load = [500–10,000[ at M6 and <500 at M12	+64	+37;+90	
Viral load = [500–10,000[ at M6 and = [500–10,000[ at M12	+23	−22;+68	
Viral load = [500–10,000[ at M6 and ≥10,000 at M12	−25	−76;+26	
Viral load ≥10,000 at M6 and <500 at M12	+87	+59;+115	
Viral load ≥10,000 at M6 and = [500–10,000[ at M12	+46	0;+93	
Viral load ≥10,000 at M6 and ≥10,000 at M12	−1	−51;+48	

* For the reference group: up to 39 year old women, starting ART up to 2003 at clinical stage WHO I,II or CDC A,B with a viral load <500 copies/mL six months after ART initiation.

ART: Antiretroviral treatment.

95% CI: 95% confidence interval.

## Discussion

Our data demonstrates that among patients initiating therapy in RLS with (mainly) an NNRTI-based regimen, CD4 cell count recovery is impaired in the presence of on-going HIV-RNA replication over an 18-month period. We were not able to find a minimal HIV-RNA threshold below which viral replication had no impact on immunological restoration. Our analysis showed an impact of HIV-RNA replication at 12 months on the direction of the CD4 cell count slope; thresholds of 10,000 or 5,000 copies were equally predictive of the direction of the CD4 cell count slope in the adjusted analysis.

Current ART guidelines recommend changing therapy when virological failure occurs [14-16]. The WHO latest recommendations reinforce the role of virological monitoring in informing the switching decision [14], a substantial change compared to the earlier versions. We used data from a multicentre cohort collaboration capturing routine data in various RLS on three continents, in which we only included the subset of patients with regular HIV-RNA and CD4 cell count monitoring. In these patients, we confirmed that the percentage of patients with short term virological success on an NNRTI-based first-line therapy was excellent [20,21].

The optimal response to low or moderate-level viremia in patients on ART in RLS has been controversial. Analyses performed in countries with regular and frequent HIV-RNA monitoring and using PI-based ARV regimens tend to suggest that the use of an HIV-RNA threshold of 5,000-10,000 copies/mL to define failure in a regimen-adherent patient with no other reasons for an elevated HIV-RNA (e.g., drug-drug interactions, poor absorption, and concurrent illness) is not associated with clinical and immunological deterioration in cohort studies [17,18,22]. There are substantial differences between patients in high-income countries and patients in RLS that prevent us from generalising findings from Western cohorts. Firstly, patients starting ART in RLS have much lower CD4 cell counts at treatment initiation when compared with high-income countries [23]. Secondly, most first-line regimens include a combination of two nucleoside analogues (NRTI) and one NNRTI, unlike high-income countries in which half of new patients are put on a boosted protease inhibitor-based ART. Finally, one might infer that immunity is affected by numerous antigenic stimulations in countries endemic for malaria or tuberculosis [24].

It is thus critical to accumulate data from countries where the majority of patients are now receiving ART, where the treatment switch is going to occur late and where first-line ARV combinations are different from the standard practice in high-income countries from where most clinical guidelines have been issued.

Because the WHO now recommends using targeted or sometimes routine use of HIV-RNA measurements in public-health oriented programs, much effort needs to go into making HIV-RNA determination available in RLS. However, the cut-off describing when to switch patients failing an NNRTI first-line regimen with few remaining therapeutic options has not been firmly established and depends on the objective: if the aim is to impact on HIV transmission or to prevent viral resistance [25-28], then viral load measurement has to be widely implemented over all ART programs and the lowest threshold must be used to inform the switching decision. However, it is critical to determine whether there is a viral threshold below which the goal of maintaining a reasonable level of CD4 cells and a good clinical outcome is achievable without switching treatment.

In this large multicohort study, we observed that in patients experiencing virological failure, the impact on immune restoration was observed after the first year of ARV treatment; CD4 cell count slope one year after ART initiation was altered by any (even low-level) HIV-RNA replication, regardless of the threshold considered (5,000 or 10000 copies/mL). By contrast, all patients who had a suppressed viral load 12 months after ART commencement experienced a rise in CD4 cell counts (Table 2).

While our study lacked the statistical power necessary to categorise what threshold would best stratify the decision for changing ART regimens, our analysis suggests that any viral replication had a deleterious impact on immune recovery in patients remaining on a failing first-line regimen.

Interestingly, patients who had residual HIV-RNA replication in the first six months on treatment, but were then virologically suppressed at M12, experienced an immune restoration with a positive CD4 cell count slope from M6 up to M18. This could suggest that routine, compared to random, viral load determination may allow for therapeutic intervention leading to successful immune restoration. Hoffmann et al. nicely showed that patients on a first line regimen in South Africa who developed viremia were successfully re-suppressed after implementing intensive adherence support [29].

The individual risk of switching treatment late after recognition of failure in first line treatment has been controversial in resource-limited settings. Seyler et al. showed in Côte d’Ivoire that patients with a detectable viral load had an increased risk of immunological failure, but there was no difference in morbidity at 18 months between the patients who switched during early regimen failure and those who remained on a failing regimen [26]. In contrast, other studies have shown that delays in switching to second-line regimens resulted in increased mortality when the failing regimen was a non-protease inhibitor-based regimen [30]. More recently, Keiser et al. demonstrated high mortality prior to and following a switch in treatment in South Africa: the cumulative mortality at 1 year was 4.2% (95% CI 2.2-7.8%) in patients who switched to a second-line regimen, 11.7% (7.3%-18.5%) in patients who remained on a failing first-line regimen, and 2.2% (1.6-3.0%) in patients on a non-failing first-line regimen (P < 0.0001). These results suggest that there are significant consequences to delayed switching [31].

Our analysis also confirmed some interesting determinants of the CD4 cell count slope; subjects with more extreme ages (below 30 years old or above 40 years old) had a better immune recovery when compared with the middle-age group. Additionally, the calendar year of starting ART and the stage at ART initiation were good predictors of early immunological outcome. Considering an HIV-RNA threshold of 10,000 copies/mL, which provided in our multivariate model the best prognostic value for immunological response, elevated HIV-RNA levels were always associated with impaired immune reconstitution.

We recognise several limitations in our analysis. Patients have been included in our study according to the existence of frequent HIV-RNA monitoring, but only a minority of patients fulfilled our criteria of biological monitoring. Selection bias is therefore possible, although we verified that none of the patients in our study had entered specific therapeutic trial protocols but were rather prescribed viral load testing during specific periods when this laboratory exam was available in the clinic. In addition, we were unable to detect differences between treatment regimens, as patients in RLS are generally prescribed with standard NNRTI-based first-line regimens. However, treatment homogeneity is helpful in controlling confounding factors related to different drug regimens. Finally, we could not consider clinical endpoints, as we had a very limited number of deaths in the selected patient population, possibly due to the short follow-up. For this reason, the clinical significance of the differences in CD4 slope reported in our analysis cannot be evaluated – although many cohorts from resource limited settings clearly assessed the relationship between AIDS-related events, non AIDS related events and death and CD4 cells [32].

## Conclusion

Our data suggest that a failing regimen with documented HIV-RNA replication should be switched onto a second-line regimen, in line with recently issued WHO guidelines requesting a decrease in the HIV-RNA threshold to inform the switching decision (from 10,000 copies in the 2006 guidelines down to 5,000 in the 2009–2010 revision of the guidelines). We recognise that immune recovery is not the only factor to consider regarding HIV disease management in the short-term after ART initiation. Whenever possible, HIV-RNA levels should be fully suppressed, not only to prevent accumulation of resistance mutations but also to prevent HIV transmission [28]. It is therefore critical to promote access to low cost, context-adapted viral load tools.

## Competing interests

We have no competing interests to declare in relation to this paper. The content of this publication is solely the responsibility of the authors and does not necessarily represent the official views of any of the institutions mentioned above.

## Authors’ contributions

AB, ES, RW, ED, EM, JM, KMEF, MS, NK, DB, PM, SVDB, and CZ contributed the necessary data from their health facilities. EB collected the data. AC, FB, FD contributed to the study design. EB and RT carried out the statistical analysis and EB, RT, AC, FB and FD interpreted the results. FD, AC and RT contributed to the study coordination. AC first drafted the manuscript. All authors had a critical revision of the manuscript for important intellectual content. All authors have read and approved the final manuscript.

## Collaborating centers

The ART-LINC collaboration of the International epidemiological Databases to Evaluate AIDS (IeDEA) is funded by the US National Institutes of Health (Office of AIDS Research and National Institute of Allergy and Infectious Diseases) and the French *Agence Nationale de Recherches sur le Sida et les hepatites virales* (ANRS). Centre de Prise en Charge de Recherches et de Formation (CEPREF)/Agence Nationale de Recherches sur le Sida et les hepatitis virales (ANRS) 1203 COTRAME Cohort (Abidjan, Côte d’Ivoire); Senegalese Antiretroviral Access Initiative (ISAARV), ANRS 1290 (Dakar, Senegal); Academic Model for the Prevention and Treatment of HIV/AIDS (AMPATH), Moi University College of Health Sciences/University of Indiana (Eldoret, Kenya); Adherence Monitoring Uganda (AMU) cohort, Makerere-University of California in San Francisco (UCSF; Kampala, Uganda); Kamuzu Central Hospital/Lighthouse Trust (Lilongwe, Malawi); Connaught Clinic (Harare, Zimbabwe); Gugulethu ART Programme, (Cape Town, South Africa); Khayelitsha ART Programme, (Cape Town, South Africa); Operational Research on ART (OPERA), Perinatal HIV Research Unit (Soweto, South Africa); Morocco Antiretroviral Treatment 14 Cohort, Centre Hospitalier Universitaire (Casablanca, Morocco); MTCT-Plus Initiative, International Center for AIDS Care and Treatment Programs, Mailman School of Public Health, Columbia University, New York, USA; Prospective Evaluation in the Use and Monitoring of Antiretrovirals in Argentina (PUMA), Buenos Aires, Argentina; South Brazil HIV Cohort (SOBRHIV), Hospital de Clinicas (Porto Alegre, Brazil); Rio de Janeiro HIV Cohort, Hospital Universitario Clementino Fraga Filho (Rio de Janeiro, Brazil); Y R Gaitonde Centre for AIDS Research and Education (YRG) Care Cohort (Chennai, India); HIV-NAT, Thai Red Cross AIDS Research Centre (Bangkok, Thailand).

## Funding/support

The ART-LINC of IeDEA Collaboration was funded by the United States National Institute of Health (NIH - Office of AIDS Research) together with the French *Agence Nationale de Recherches sur le Sida et les hépatites virales* (ANRS – grants 12101 and 12138). The IeDEA network is now funded through collaborative agreements by the National Cancer Institute (NCI), the Eunice Kennedy Shriver National Institute of Child Health and Human Development (NICHD) and the National Institute of Allergy And Infectious Diseases (NIAID).

## The ART-LINC of IeDEA Central Coordinating Team (in alphabetical order)

Eric Balestre, Martin Brinkhof, François Dabis (principal investigator), Matthias Egger (principal investigator), Claire Graber, Beatrice Fatzer, Olivia Keiser, Charlotte Lewden, Mar Pujades, Mauro Schechter (principal investigator).

## Pre-publication history

The pre-publication history for this paper can be accessed here:

http://www.biomedcentral.com/1471-2334/12/147/prepub

## References

[B1] Pujades-Rodriguez M O’Brien D Humblet P Calmy A Second-line antiretroviral therapy in resource-limited settings: the experience of Medecins Sans Frontieres AIDS 2008 22 1305 1312 10.1097/QAD.0b013e3282fa75b9 18580610

[B2] Keiser O Orrell C Egger M Swiss HIV Cohort Study (SHCS) and the International Epidemiologic Databases to Evaluate AIDS in Southern Africa (IeDEA-SA) Public-health and individual approaches to antiretroviral therapy: township South Africa and Switzerland compared PLoS Med 2008 5 e148 Erratum in: PLoS Med. 2008; 9:e195 10.1371/journal.pmed.0050148 18613745 PMC2443185

[B3] Keiser O Tweya H Boulle A ART-LINC of IeDEA Study Group Switching to second-line antiretroviral therapy in resource-limited settings: comparison of programmes with and without viral load monitoring AIDS 2009 23 1867 74 19531928 10.1097/QAD.0b013e32832e05b2 PMC2956749

[B4] Renaud-Théry F Nguimfack BD Vitoria M Lee E Graaff P Samb B Perriëns J Use of antiretroviral therapy in resource-limited countries in 2006: distribution and uptake of first- and second-line regimens AIDS 2007 Suppl 4 S89 95 17620758 10.1097/01.aids.0000279711.54922.f0

[B5] UNAIDS epidemic update 2009 Available from http://www.unaids.org/en/Dataanalysis/Epidemiology/2009AIDSEpidemicUpdate/ (accessed on March 7th, 2011)

[B6] World Health Organization Antiretroviral therapy for HIV Infection in adults and adolescents in resource-limited settings: towards universal access. Recommendations for a public health approach Available from http://www.who.int/hiv/mediacentre/universal_access_progress_report_en.pdf (accessed August 4th, 2011)

[B7] Moore DM Mermin J Awor A Yip B Hogg RS Montaner JS Performance of immunologic responses in predicting viral load suppression: implications for monitoring patients in resource-limited settings J Acquir Immune Defic Syndr 2006 43 436 439 10.1097/01.qai.0000243105.80393.42 17019367

[B8] Koenig SP Kuritzkes DR Hirsch MS Monitoring HIV in developing countries BMJ 2006 332 602 604 10.1136/bmj.332.7541.602 16528087 PMC1397781

[B9] Mermin J Ekwaru JP Were W Utility of routine viral load, CD4 cell count, and clinical monitoring among HIV-infected adults in Uganda: A randomized trial BMJ 2011 343 d6792 10.1136/bmj.d6792 22074711 PMC3213241

[B10] Mugyenyi P Walker AS Hakim J Munderi P Gibb DM DART Trial Team Routine versus clinically driven laboratory monitoring of HIV antiretroviral therapy in Africa (DART): a randomized non-inferiority trial Lancet 2010 375 123 31 20004464 10.1016/S0140-6736(09)62067-5 PMC2805723

[B11] Chang LW Harris J Humphreys E Optimal monitoring strategies for guiding when to switch first-line antiretroviral therapy regimens for treatment failure in adults and adolescents living with HVI in low-resource settings Cochrane Database Syst Rev 2010 4 CD008494 20393969 10.1002/14651858.CD008494 PMC13574227

[B12] Jourdain G Ngo-Giang-Huong N PHPT-3: a randomized clinical trial comparing CD4 vs. viral load ART monitoring/Switching strategies in Thailand18th Conference on retroviruses and opportunistic infections, Boston 2011 Abstract 44

[B13] Kouanfack C Laurent C Laborde-Balen G for the Stratall ANRS 12110/ESTHER Study Group HIV viral load, CD4 cell count, and clinical monitoring s. clinical monitoring alone for ART in rural hospitals in Cameroon: stratall ANRS 12110/ESTHER trial, a randomized non inferioirity trial18th Conference on retroviruses and opportunistic infections, Boston 2011 Abstract 45 LB

[B14] World Health Organization Antiretroviral therapy for HIV infection in adults and adolescents. Recommendations for a public health approach (2010 version) Available from http://www.who.int/hiv/pub/arv/adult2010/en/index.html (accessed on 4th August, 2011) 23741771

[B15] Guidelines for the Use of Antiretroviral Agents in HIV-1-Infected Adults and Adolescents - January 10, 2011 available from http://aidsinfo.nih.gov/contentfiles/AdultandAdolescentGL.pdf (accessed on March 7th, 2011)

[B16] European AIDS Clinical Society guidelines, version 6.0, October 2011 European AIDS Clinical Society guidelines, version 6.0, October 2011 accessed on April 2d, 2012 http://www.europeanaidsclinicalsociety.org/images/stories/EACS-Pdf/eacsguidelines-v6_english.pdf

[B17] PLATO collaboration Predictors of trends in CD4-positive T cell count and mortality among HIV-1 infected individuals with virological failure to all three antiretroviral-drug classes Lancet 2004 364 51 62 10.1016/S0140-6736(04)16589-6 15234856

[B18] Murri R Cozzi-Perri A Cicconi P for the Icona study group Is moderate HIV viremia associated with a higher risk of clinical progression in HIV positive people treated with HAART? Evidence from the Italian cohort of antiretroviral naïve patients study J Acquir Immune Defic Syndr 2006 41 23 30 10.1097/01.qai.0000188337.76164.7a 16340469

[B19] Dabis F Balestre E Braitstein P Antiviral Therapy in Lower Income Countries (ART-LINC) Study Group. Cohort Profile: Antiretroviral Therapy in Lower Income Countries (ART-LINC): international collaboration of treatment cohorts Int J Epidemiol 2005 34 979 86 16157617 10.1093/ije/dyi164

[B20] Nachega JB Hislop M Nguyen H Antiretroviral therapy adherence, virologic and immunologic outcomes in adolescents compared with adults in southern Africa Acquir Immune Defic Syndr 2009 51 65 71 10.1097/QAI.0b013e318199072e PMC2674125 19282780

[B21] Bourgeois A Laurent C Mougnutou R Field assessment of generic antiretroviral drugs: a prospective cohort study in Cameroon Antivir Ther 2005 10 335 41 15865228

[B22] Cozzi Lepri A Phillips AN d'Arminio Monforte A When to start highly active antiretroviral therapy in chronically HIV-infected patients: evidence from the ICONA study AIDS 2001 15 983 90 10.1097/00002030-200105250-00006 11399980

[B23] ART LINC collaboration and ART-CC collaboration Mortality of HIV1-infected patients in the first year of antiretroviral therapy: comparison between low-income and high-income countries Lancet 2006 367 817 824 16530575 10.1016/S0140-6736(06)68337-2

[B24] Redd AD Dabitao D Bream JH Microbial translocation, the innate cytokine response, and HIV-1 disease progression in Africa Proc Natl Acad Sci U S A 2009 106 6718 23 10.1073/pnas.0901983106 19357303 PMC2667149

[B25] Cozzi-Lepri A Phillips AN Martinez-Picado J EuroSIDA Study Group Rate of accumulation of thymidine analogue mutations in patients continuing to receive virologically failing regimens containing zidovudine or stavudine: implications for antiretroviral therapy programs in resource-limited settings J Infect Dis 2009 200 687 97 10.1086/604731 19604043

[B26] Seyler C Adje-Toure C Messou E Impact of genotypic drug resistance mutations on clinical and immunological outcomes in HIV-infected adults on HAART in West Africa AIDS 2007 21 1157 1164 10.1097/QAD.0b013e3281c615da 17502726 PMC2486349

[B27] Phillips AN Pillay D Garnett G Effect on transmission of HIV-1 resistance of timing of implementation of viral laod monitoring to determine switches from first to second-line antiretroviral regimens in resource-limited settings AIDS 2011 25 843 850 10.1097/QAD.0b013e328344037a 21192233

[B28] Cohen MS Chen YQ McCauley M HPTN 052 Study Team Prevention of HIV-1 infection with early antiretroviral therapy N Engl J Med 2011 365 493 505 10.1056/NEJMoa1105243 21767103 PMC3200068

[B29] Hoffmann CJ Charalambous S Sim J Viremia, resuppression, and time to resistance in human immunodeficiency virus (HIV) subtype C during first-line antiretroviral therapy in South Africa Clin Infect Dis 2009 49 1928 1935 10.1086/648444 19911963 PMC2789416

[B30] Petersen ML van der Laan MJ Napravnik S Long-term consequences of the delay between virologic failure of highly active antiretroviral therapy and regimen modification AIDS 2008 22 2097 2106 10.1097/QAD.0b013e32830f97e2 18832873 PMC2597677

[B31] Keiser O Tweya H Braitstein P Mortality after failure of antiretroviral therapy in sub-Saharan Africa Trop Med Int Health 2010 15 251 8 10.1111/j.1365-3156.2009.02445.x 20003034 PMC2873139

[B32] Anglaret X Minga A Gabillard D AIDS and non-AIDS morbidity and mortality across the spectrum of CD4 cell counts in HIV-infected adults before starting antiretroviral therapy in Cote d'Ivoire Clin Infect Dis 2012 5 714 23 22173233 10.1093/cid/cir898 PMC3275759

